# Generation and immunogenicity assessment of ELPylated virus-like particles of porcine circovirus type 2

**DOI:** 10.1186/s12985-020-01346-6

**Published:** 2020-06-09

**Authors:** Yangyang Li, Yajie Wang, Jian Cheng, Xiaohui Zhou, Huipeng Lu, Xinyu Zhang, Xiaoli Xia, Huaichang Sun

**Affiliations:** 1 College of Veterinary Medicine, Jiangsu Co-innovation Center for Prevention and Control of Important Animal Infectious Diseases and Zoonoses, Yangzhou University, Yangzhou, 225009 China; 2 Jiangsu Key Laboratory for High-Tech Research and Development of Veterinary Biopharmaceuticals, Jiangsu Agri-animal Husbandry Vocational College, Taizhou, 225300 China

**Keywords:** PCV2 cap protein, ELP fusion expression, VLP preparation, Immunogenicity comparison

## Abstract

**Background:**

Porcine circovirus type 2 (PCV2) is an economically important pathogen affecting swine industry worldwide. The production of current PCV2 vaccines is time-consuming and expensive. Elastin-like polypeptides (ELP) undergo temperature-dependent inverse phase transition and ELPylated proteins can be purified simply by inverse transition cycling (ITC).

**Methods:**

The Cap protein of PCV2b, together with the virus neutralizing (VN) epitopes of PCV2a, PCV2d and PCV2e, was expressed in *E. coli* as an ELPylated protein, and purified by ITC in the presence of mild detergents. For the control purpose, the Cap protein was also expressed as a His-tagged protein and purified by nickel affinity chromatography. The formation of ELPylated VLP (ELP-VLP) and His-tagged VLP (VLP) was revealed by transmission electron microscopy. Mice were immunized two times with the two forms of VLP and the antigen-specific IgG antibody, VN antibody, cytokine responses and immunoprotection against PCV2 challenge were compared.

**Results:**

ELPylated Cap protein was expressed as a soluble protein and purified to 94.3% purity by ITC in the presence of 1% Triton X-100 and 0.5 M urea. His-tagged Cap fusion protein was expressed as insoluble inclusion bodies and purified to 90% purity under denatured conditions. The two purified fusion proteins assembled into VLP with similar morphology. Compared to immunization with VLP, immunization with ELP-VLP induced significantly (*p* < 0.01) stronger VN antibody response and slightly (*p* < 0.05) stronger Cap-specific IgG antibody response, cytokine production and immunoprotection against PCV2 challenge.

**Conclusion:**

A novel ELPylation platform for easy preparation of PCV2 VLP was established and the prepared ELP-VLP was more immunogenic than VLP. The ELPylation technology could be used for other VLP preparation and the prepared ELP-VLP could be developed as a novel PCV2 subunit vaccine.

## Background

Porcine circovirus type 2 (PCV2) is the primary causative agent of several syndromes collectively known as porcine circovirus-associated disease (PCVAD) [1, 2]. Vaccination is an effective and economical method for the control of PCV2 infection. Currently, at least five commercial PCV2 vaccines are available in the international market, including the inactivated vaccine, capsid protein-based vaccines and chimerical PCV1/2 vaccine [3]. The inactivated vaccine is produced commonly in PK-15 cells with low yield due to poor propagation of PCV2 in PK-15 cells [4]. The capsid protein-based virus-like particle (VLP) vaccine can be produced in *E. coli* or baculovirus system, which requires expensive ultracentrifugation or chromatography for purification [5, 6]. Therefore, reduction of PCV2 vaccine production cost is a key priority for veterinary research.

Elastin-like polypeptides (ELP) are derivatives of tropoelastin with the pentapeptide (Val-Pro-Gly-Xaa-Gly) repeats, where Xaa can be any amino acid except proline. ELP have a unique property, inverse phase transition, which allows temperature-dependent reversible change from soluble monomers to insoluble aggregates [7, 8]. Fusion of ELP with a target protein at the genetic level is now termed ELPylation, which has been exploited for several biomedical applications, such as recombinant protein purification [9], drug delivery [10] and protein half-life extension [11]. Like ELP, ELPylated proteins can be purified by inverse transition cycling (ITC) with the advantages of simplicity and low cost. Since they are derived from tropoelastin, ELP are biocompatible, non-toxic and non-immunogenic, making ELPylated proteins suitable for in vivo applications [12]. More recently, ELPylation has been used to improve the immunogenicity of influenza virus M protein [13] and hemagglutinin [14].

In the present study, we explored the feasibility of ELPylation technology for simple purification and immunogenicity improvement of PCV2 VLP. The Cap protein of PCV2b, together with the virus neutralizing (VN) epitopes of PCV2a, PCV2d and PCV2e, was expressed in *E. coli* as an ELPylated protein, and purified to a high purity with modified ITC. For the control purpose, the Cap protein was also expressed as a His-tagged protein and purified by nickel affinity chromatography. Both ELPylated and His-tagged Cap proteins assembled into VLP with similar morphology. Immunization of mice showed that ELPylated VLP was more immunogenic than His-tagged VLP. To our knowledge, this is the first study to demonstrate that ELPylation can be used for VLP preparation and immunogenicity improvement.

## Materials and methods

### Vector construction

**Fig. 1:**
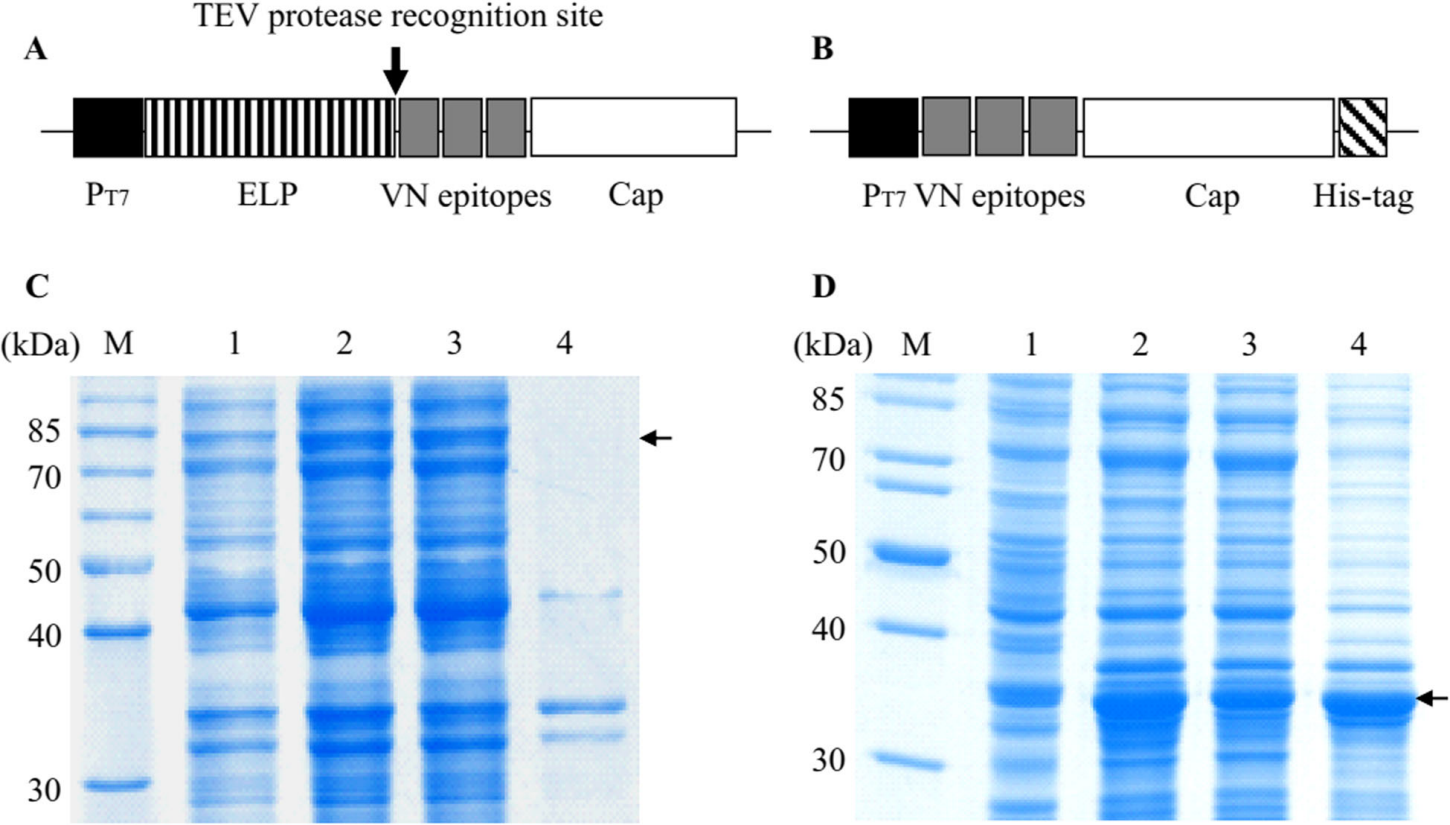
Expression of ELP-Cap and Cap-His fusion proteins in *E. coli***. a**, **b** The schematic structures of pELP-Cap and pET-Cap vectors. The coding sequences for ELP, His-tag, Cap protein of PCV2b and the VN epitopes of PCV2a, PCV2d and PCV2e are indicated. The expression of fusion sequence was under the control of T7 promoter (PT7). **c**, **d** SDS-PAGE analysis of ELP-Cap and Cap-His fusion protein expression. The expression of two fusion proteins was induced for 24 h at 20 °C with 0.2 mM IPTG. After sonication treatment, the total cell lysates of recombinant *E. coli* before IPTG induction (1), after IPTG induction (2), supernatants (3) and pellets (4) of centrifuged cell lysates were analyzed by 12% SDS-PAGE. M indicates protein molecular mass marker. The small arrows indicate ELP-Cap and Cap-His fusion proteins

ELP fusion expression vector pET-ELP was constructed by cloning ELP coding sequence into pET-30a (+) vector (Novagen, USA) with *Nde*I and *Sac*I digestion [15]. The coding sequences for the Cap protein of PCV2b (GenBank accession: GQ359004) and the VN epitopes [16] of PCV2a (GenBank accession: GQ359003), PCV2d (GenBank accession: GU001710) and PCV2e (GenBank accession: GU001709) were adapted to *E. coli* codon usage using JAVA Codon Adaption Tool [17]. The synthetic sequence, with a tobacco etch virus (TEV) protease recognition signal introduced at the 5′ end, was cloned into pET-ELP vector with *Hin*dIII and *Xho*I digestion. For the comparison purpose, the synthetic sequence was amplified by PCR using the forward primer (5′-CAGTACATCAAAGCTAACTC-3′) and the reverse primer (5′-CGGGTTCAGCGGCGGGTCTTT-3′), and cloned into pET-30a vector with *Nde*I and *Sal*I digestion. The recombinant vectors were called pELP-Cap and pET-Cap (Fig. 1), and the expressed proteins were called ELP-Cap and Cap-His, respectively.

### Protein expression

Both pELP-Cap and pET-Cap vectors were transformed individually into BL21 (DE3) *E. coli*. After growth for 5 h in 2 × YT medium (10 g yeast extract, 16 g tryptone, 5 g NaCl/l, pH 7.2) containing kanamycin (50 μg/ml), the expression of the two fusion proteins was induced for 24 h at 20 °C with 0.2 mM IPTG (isopropyl β-D-thiogalactoside). After 10-min centrifugation at 5000 g, the bacterial pellets were suspended in a lysis buffer (50 mM Tris-HCL, pH 8.0, 2 mM EDTA, 0.5%Triton X-100, 1 mM DTT, 5% glycerol), and disrupted two times at 1300 bar using High Pressure Cell Disruptor (JNBIO, China). After centrifugation for 15 min at 14,000 g, the supernatants were collected for recombinant protein purification.

### Protein purification

ELPylated Cap protein was purified by ITC as previously described [18] with slight modification. Briefly, after determination of the transition temperature and salt (NaCl) concentration, ITC was performed in the presence of different concentrations of Triton X-100 and/or different concentrations of urea. After 5-min centrifugation at 14,000 g at room temperature, the protein pellet was washed with 1%Triton X-100 and 0.5 M urea in 3 M NaCl. The residual endotoxin was removed by one round of Triton X-114 isothermal extraction as previously described [19]. His-tagged Cap protein was purified with His-Tagged Protein Purification Kit (CWBIO, China) under denatured conditions by following the manufacturer’s instruction. Finally, the two purified proteins were dialyzed in VLP assembling buffer (50 mM Na_2_HPO_4_, 50 mM NaH_2_PO_4_, 500 mM NaCl, 1 mM EDTA, 0.03% Tween 80, pH 6.5). After 5-min centrifugation at 12,000 g, the supernatants were collected for further study.

### ELP tag cleavage

The recombinant TEV protease was expressed in *E. coli* as a fusion protein with self-aggregating peptide ELK16 and purified by centrifugation in the presence of 0.5% Triton X-100 as previously described [19]. The purified ELPylated Cap protein (100 μg) was digested overnight with the recombinant protease (30 μg) as previously described [19]. After digestion, the active aggregates of TEV protease were removed by centrifugation and the cleaved ELP tag was removed by one round of ITC as described.

### Transmission electron microscopy

Both ELPylated and His-tagged Cap proteins (25 μg) were absorbed onto copper grids (400 meshes) for 2.5 min at room temperature. After drying gently with filter paper, the grids were stained with 3% phosphotungstic acid for 2.5 min. The excess liquid was removed and the samples were observed under transmission electron microscope (Philips, Tecnai 12, Netherland) at an acceleration voltage of 75 kV.

### Western blotting

Both ELPylated VLP (ELP-VLP) and His-tagged VLP (VLP) were separated on 12% SDS-PAGE and transferred to nitrocellulose membrane (Merck, USA) using a Mini-Protean® Tetra Cell (Bio-Rad, USA) by following the manufacturer’s instruction. The membrane was blocked for 2 h at 37 °C with 5% skim milk powder in PBST (0.1% Tween 20 in PBS), and incubated for 1 h at 37 °C with home-made pig anti-PCV2 serum (1:500). After three-time washing in PBST, the membrane was incubated for 30 min at 37 °C with DeLyght^800^-labeled goat anti-pig IgG (1:10,000) (KPL, USA). The hybridization signal was scanned using Infrared Imaging System (Odyssey, USA) at 800 nm by following the manufacturer’s instruction.

### Animal immunization and virus challenge

Six-week-old BALB/c mice were purchased from the Center of Comparative Medicine, Yangzhou University. Eighteen mice were randomly divided into three groups (6 for each group). The mice in groups 1 and 2 were immunized intramuscularly with 200 μl (50 μg) of ELP-VLP or VLP without use of adjuvant. The mice in group 3 were injected with the same volume of PBS as the negative control. The primarily immunized mice were boosted with the same dose of antigen at day 14 post immunization (dpi). The blood samples were collected at 7, 14, 21 and 28 dpi for antibody detection. Three mice from each group were sacrificed at 28 dpi for splenocyte isolation and cytokine detection. The remaining three mice in each group were challenged intraperitoneally with 2 × 10^3^ TCID_50_ (50% tissue culture infectious dose) of PCV2b. On days 7 and 14 post challenge (dpc), the serum samples were collected for PCV2 DNA detection.

### Indirect ELISA

Bottom-flattened 96-well microplates were coated overnight at 4 °C with His-tagged Cap protein (5 μg/ml) in 0.1 M carbonate buffer (pH 9.6). After blocking for 1 h at 4 °C with 5% skim milk powder in PBST (0.05% Tween 20 in PBS), the plates were incubated for 1 h at 37 °C with the serum samples (1:100 in PBST). After three-time washing with PBST, HRP (horse radish peroxidase)-conjugated goat anti-mouse IgG (1:10,000 in PBST) (Sangon Biotech, China) was added and incubated for 1 h at 37 °C. After washing again, HRP signal was developed for 20 min with TMB (tetramethylbenzidine) substrate and OD_450_ values were measured on an ELISA reader.

### VN antibody test

The VN antibody test was performed on 96-well microtitration plates using PK-15 cells as the indicator as previously described [20] with slight modification. Briefly, 100 μl (4 × 10^5^) of cells was seeded to each well and grown for 24 h at 37 °C in DMEM containing 10% FBS (fecal bovine serum). Serum samples were heat-inactivated for 30 min at 56 °C, and serially diluted twofold up to 1:512. Each dilution (100 μl) and an equal volume of PCV2b (200 TCID_50_) were added and incubated for 60 min at 37 °C. After washing with PBS, 200 μl of DMEM containing 2% FBS was added, and incubated for 24 h at 37 °C. After fixing with 80% cold acetone, immunofluorescence was performed using pig anti-PCV2 serum (1:500) as the first antibody and FITC-conjugated goat anti-pig IgG (1:1000) (Sigma, USA) as the second antibody. VN antibody titers were expressed as the highest dilutions in which no or higher than 80% reduction of virus replication was detected as compared to the virus control.

### Cytokine detection

Each spleen sample from the immunized mouse was pushed through 1-ml syringe and centrifuged for 10 min at 1500 g. The cell pellet was suspended with 500 μl of PBS and mixed with 3 ml of red blood cell lysis buffer (Beyotime Biotechnology, China). After 5-min incubation at room temperature, the splenocytes were collected by 10-min centrifugation at 1500 g, washed two times with PBS and cultured (5 × 10^6^ cells/ml) overnight in RPMI 1640 medium (Hyclone, USA) supplemented with 10% FBS. After stimulation for 72 h at 37 °C with His-tagged Cap protein (10 μg/ml) or Con A (10 μg/ml) (Sigma, USA), the cell culture was centrifuged for 10 min at 3000 g, and the supernatant was collected for cytokine detection. Interleukin 4 (IL-4), tumor necrosis factor-α (TNF-α) and interferon-γ (IFN-γ) were detected in triplicates using the ELISA Kits (Boster Bio, China) by following the manufacturer’s instruction.

### Quantitative PCR

The serum samples from immunized and PCV2-challenged mice were extracted using MiniBEST Viral RNA/DNA Extraction Kit (TaKaRa, China) by following the manufacturer’s instruction. PCV2 DNA copies were detected in triplicates using the *Cap*-specific forward primer (5-AAGGGCTGGGTTATGGTATG-3) and reverse primer (5-GAGTGGGCTCCAGTGCTGTTA-3). The quantitative PCR (20 μl) was performed using 2 μl of DNA template and SYBR Premix Ex Taq™II (TaKaRa, China) by following the manufacturer’s instruction. The standard curve was generated using pMD-18 T vector (TaKaRa, China) containing the PCR-amplified Cap gene segment.

### Statistical analysis

Statistical analysis was performed using SPSS Statistics 22. The results were considered to be statistically significant at *p* < 0.05 or extremely significant at *p* < 0.01. For each separate set of data, at least three independent assays were performed and the results were represented as mean ± standard deviation (SD).

## Results

### Vector construction and protein expression

To enhance the immunogenicity of PCV2b Cap protein, the coding sequence was fused with the coding sequences for the VN epitopes of PCV2a, 2d and 2e. To facilitate the protein purification, the synthetic sequence was cloned into pET-ELP vector and expressed as an ELPylated protein (Fig. 1a). For the comparison purpose, the synthetic sequence was cloned into pET-30a vector (Fig. 1b) and expressed as a His-tagged protein.

To facilitate the soluble protein expression, the expression of two fusion proteins was induced slowly at 20 °C with 0.2 mM IPTG. SDS-PAGE analysis showed that an expected 85-kDa protein was expressed in pELP-Cap recombinant *E. coli*, which was present in the soluble fraction of centrifuged bacterial lysate (Fig. 1c). An expected 35-kDa protein was expressed in pET-Cap recombinant *E. coli*, which was present mainly in the insoluble fraction of centrifuged bacterial lysate (Fig. 1d).

### ELPylated cap fusion protein was purified to high purity by modified ITC

**Fig. 2:**
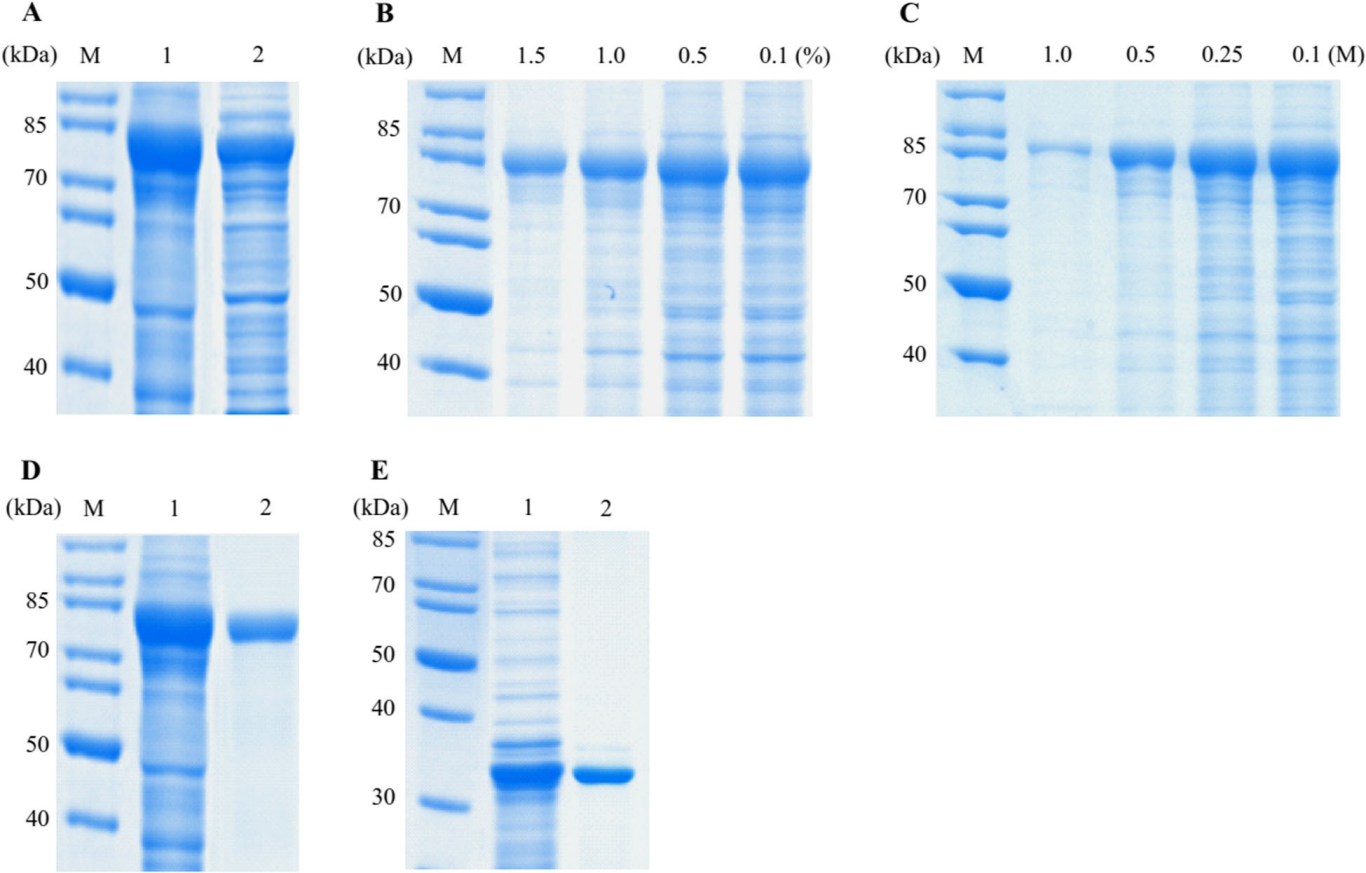
Purification of ELP-Cap and Cap-His fusion proteins. **a** The total cell lysate of pELP-Cap recombinant *E. coli* (1) and ELP-Cap fusion protein (2) purified by ITC. **b** Purification of ELP-Cap protein by ITC in the presence of different concentrations of Triton X-100. **c** Purification of ELP-Cap protein by ITC in the presence of different concentrations of urea. **d** The total cell lysate of pELP-Cap recombinant *E. coli* (1) and ELP-Cap protein (2) purified by ITC in the presence of 1% Triton X-100 and 0.5 M urea. **e** The total cell lysate of pET-Cap recombinant *E. coli* (1) and Cap-His fusion protein (2) purified by nickel affinity chromatography. All samples were analyzed by 12% SDS-PAGE

The transition temperature of ELPylated Cap protein was 26 °C in 3 M NaCl. After one cycle of ITC, SDS-PAGE analysis showed that the Cap protein was purified to less than 50% purity (Fig. 2a). Then, ITC was performed in the presence of different concentrations of Triton X-100 or urea. SDS-PAGE analysis showed that the purity of ELPylated Cap protein could be significantly improved by including 1% Triton X-100 (Fig. 2b) or 0.5 M urea into ITC (Fig. 2c) without significant loss of protein yield. Finally, the ITC was performed in the presence of 1% Triton X-100 and 0.5 M urea. SDS-PAGE analysis showed that ELPylated Cap protein was purified to 94.3% purity with the modified ITC (Fig. 2d). His-tagged Cap protein was purified to 90.0% purity using nickel affinity chromatography under denatured condition (Fig. 2e). The performances of two protein purification methods are summarized in Table 1.

**Table 1:** Comparison of purification performances between inverse transition cycling and affinity chromatography

Protein	Expression (mg/l)	Purity (%)	Recovery (%)	Yield (mg/l)	Purification time (h)
ELP-Cap	56.16	94.3	74.6	41.91	2.5
Cap-His	49.37	90	60.77	30	23

### ELP tag could not be cleaved from ELPylated cap protein

After overnight digestion, the active aggregates of TEV protease were removed by centrifugation and the cleaved ELP tag was removed by an additional round of ITC. SDS-PAGE analysis showed that the ELP tag could not be cleaved from ELPylated Cap protein.

### Both ELPylated and his-tagged cap proteins assembled into VLPs

**Fig. 3:**
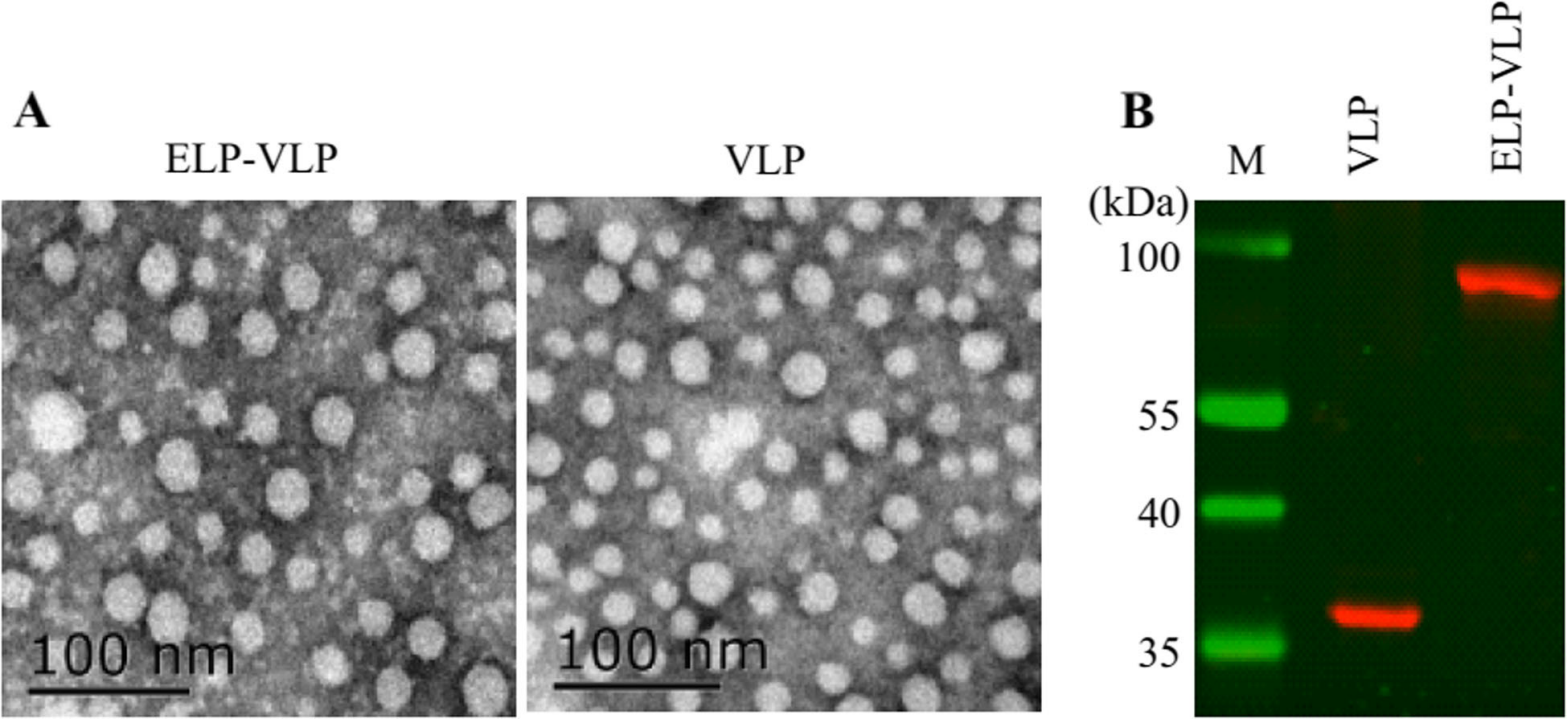
Characterization of two different forms of PCV2 VLP. **a** Transmission electron microscopy of ELP-VLP and VLP. **b** Western blotting of ELP-VLP and VLP using pig anti-PCV2 serum as the first antibody and DeLyght^800^-labeled goat anti-pig IgG as the second antibody

After dialysis in VLP assembling buffer, TEM analysis showed that both ELPylated and His-tagged Cap proteins assembled into VLPs (ELP-VLP or VLP hereafter) with diameter ranging from 15 to 20 nm (Fig. 3a). Except the slightly rough surface, the morphology of ELP-VLP was almost identical to that of VLP (Fig. 3b). Western blotting analysis showed that two forms of VLPs reacted positively with pig anti-PCV2 serum (Fig. 3c), suggesting the correct conformation of two Cap protein VLPs.

### ELP-VLP induced stronger humoral response than VLP

**Fig. 4:**
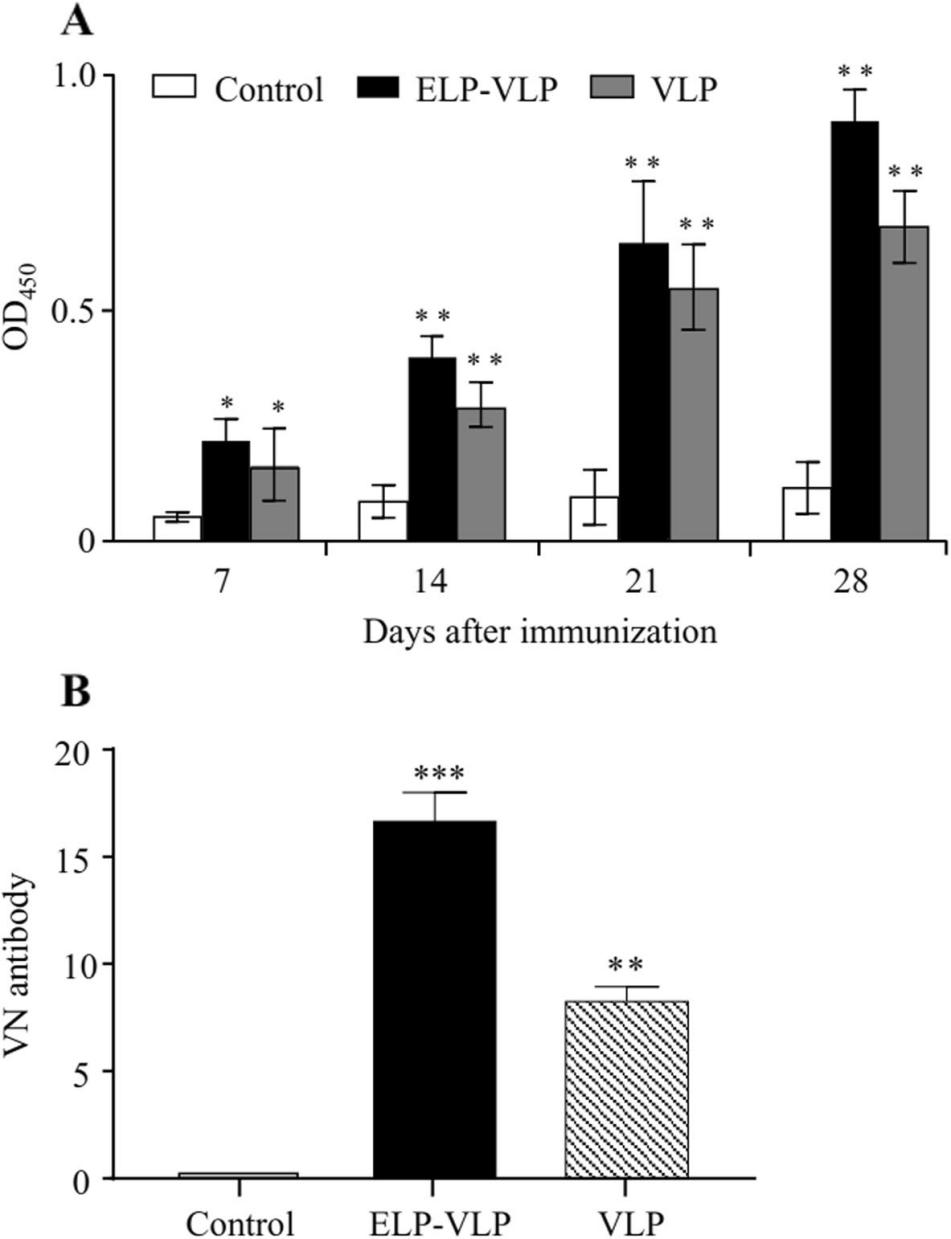
Comparison of the antibody responses after immunization with two different forms of VLPs**.** Mice were immunized two times with the same dose of ELP-VLP or VLP without use of adjuvant. **a** The serum samples were collected from the immunized and control mice at indicated time and analyzed for the Cap-specific IgG antibody. **b** The serum samples were collected from the immunized and control mice at 28 dpi and analyzed for PCV2 VN antibody. *, ** and *** indicate *p* < 0.05, *p* < 0.01 and *p* < 0.001 as compared with the control group

As compared to the control mouse serum, a significantly (*p* < 0.05) higher level of Cap-specific IgG antibody was detected in ELP-VLP or VLP immune serum as early as 7 dpi (Fig. 4a). From 14 dpi, the IgG antibody titers of the two vaccination groups, but not the control group, increased rapidly and reached to the highest levels by 28 dpi. For the inter-group comparison, the IgG antibody titer in ELP-VLP immune serum was slightly higher than that in VLP immune serum without significant difference (Fig. 4a).

Three mice from each group were sacrificed at 28 dpi and the serum samples were assayed for VN antibody against PCV2. No VN antibody was detected in the control mouse serum. In contrast, a significantly (*p* < 0.01) amount of VN antibody was detected in ELP-VLP immune serum (1:16.8) or VLP immune serum (1:8.4). For the inter-group comparison, the average VN antibody titer in ELP-VLP immune serum was significantly (*p* < 0.01) higher than that in VLP immune serum (Fig. 4b).

### ELP-VIP induced stronger cytokine response than VLP

**Fig. 5:**
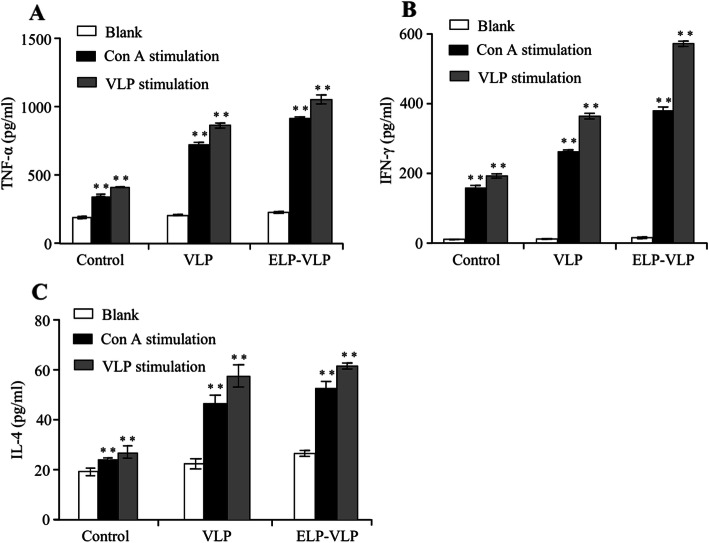
Comparison of cytokine responses after immunization with two different forms of VLPs. Three mice from each group were sacrificed at 28 dpi for splenocyte isolation. After 72-h stimulation with VLP or Con A, the splenocyte culture media were assayed for three cytokines using commercial ELISA kits. ** indicates *p* < 0.01 as compared with the control group

Three mice from each group were sacrificed at 28 dpi and the splenocytes were isolated for cytokine detection. After 72-h stimulation with VLP, the cell culture media were assayed for TNF-α, IFN-γ and IL-4. As compared with the control group, a significant (*p* < 0.01) amount of TNF-α was detected in ELP-VLP (1100 pg/ml) or VLP (870 pg/ml) vaccination group (Fig. 5a). Similarly, a significant (*p* < 0.01) amount of IFN-γ was detected in ELP-VLP (570 pg/ml) or VLP (360 pg/ml) vaccination group (Fig. 5b). Finally, a significant (*p* < 0.01) amount of IL-4 was detected in ELP-VLP (620 pg/ml) or VLP (580 pg/ml) vaccination group (Fig. 5c). For the inter-group comparison, the three cytokine responses of ELP-VLP vaccination group were slightly higher than that of VLP vaccination group without significant difference. The VLP-stimulated cytokine responses were slightly stronger than that stimulated with Con A, suggesting the specificity of three cytokine responses after ELP-VLP or VLP immunization.

### ELP-VLP induced stronger immunoprotection than VLP

**Fig. 6:**
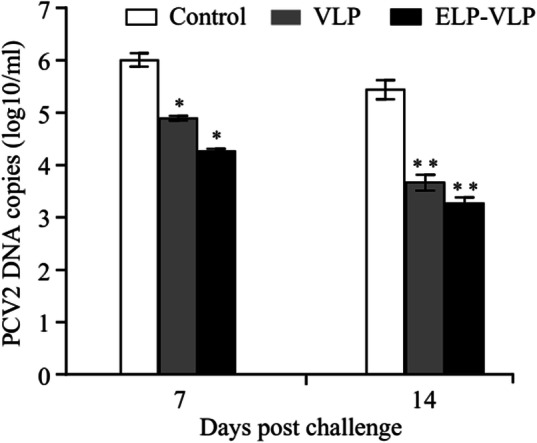
Comparison of immunoprotection of two different forms of VLP against PCV2 challenge**.** After two-time immunization with ELP-VLP or VLP, three mice from each group were challenged with PCV2b at 28 dpi. The serum samples were collected from the immunized and control mice at indicated times and assayed for PCV2 DNA copies by quantitative PCR. * and ** indicate *p* < 0.05 and *p* < 0.01 as compared with the control group

The three remaining mice in each group were challenged with PCV2 at 28 dpi and the serum samples were assayed for the viral DNA copies by quantitative PCR. At 7 dpc, the viral DNA copy number in ELP-VLP (4.21 log10/ml) or VLP (4.98 log10/ml) immune serum was significantly (*p* < 0.05) lower than that (6.01 log10/ml) in the control serum (Fig. 6). By 14 dpc, the viral DNA copy number in ELP-VLP (3.21 log10/ml) or VLP (3.72 log10/ml) immune serum was extremely significantly (*p* < 0.01) lower than that (5.52 log10/ml) in the control serum (Fig. 6). For the inter-group comparison, the viral DNA copy number in ELP-VLP immune serum was slightly lower than that in VLP immunization serum without significant difference.

## Discussion

ELP can undergo temperature-dependent inverse phase transition from soluble monomers to insoluble aggregates, and thus ELPylated proteins can be purified simply by ITC [7, 8]. The primary objective of this study was to explore the feasibility of ELPylation technology for preparation of PCV2 VLP vaccine. To this end, the Cap protein of PCV2b, together with the VN epitopes of PCV2a, PCV2d and PCV2e, was expressed in *E. coli* as an ELPylated or His-tagged protein. For ITC to be useful, ELPylated Cap protein must be expressed as a soluble protein. Therefore, the expression of the two fusion proteins was induced slowly at low temperature to facilitate soluble protein expression. As expected, ELPylated Cap protein was expressed as a soluble protein. In contrast, His-tagged Cap protein was expressed as insoluble inclusion bodies under the same conditions. This data suggests that the ELP tag could improve the solubility of recombinant Cap protein of PCV2. After one cycle of ITC, however, the purity of purified ELPylated Cap protein was less than 50%, which could not be improved by repeated ITC. Since ELP-mediated ITC can be performed in the presence of low concentrations of mild detergents [21], we modified the ITC by inclusion of 1% Triton X-100 and 0.5 M urea. By using the modified ITC, ELPylated Cap protein was purified to more than 90% purity, which was comparable to that of His-tagged Cap protein purified by nickel affinity chromatography.

After obtaining the purified fusion protein, we tried to cleave ELP tag from ELPylated Cap protein with recombinant TEV protease. Unexpectedly, ELP tag was unable to be cleaved from ELPylated Cap protein. To find the possible reason for the incapability of ELP tag cleavage, we analyzed the structure of ELPylated Cap protein by TEM. As expected, ELPylated Cap protein assembled into VLP with morphology similar to VLP. This data confirmed that ELP do not interfere with recombinant protein folding [22]. Therefore, the incapability of ELP tag cleavage could be explained by burring of TEV protease recognition site inside of ELP-VLP. Since ELP can improve the immunogenicity of recombinant protein vaccines by protecting against antigen degradation [13, 14], the cleavage of ELP tag from ELP-VLP was unnecessary.

Currently, there is no consensus for PCV2 vaccine evaluation in animal models. Although some research groups have reported that mouse models provide only limited utility in understanding of PCVAD, mouse models have been used widely for elucidation of in vivo behaviors of the virus-host interaction and PCV2 vaccine immunogenicity [23]. Therefore, in this study we compared the immunogenicities between ELP-VLP and VLP using a mouse model. After immunization with ELP-VLP or VLP, the Cap-specific IgG antibody response was detectable as early as 7 dpi, which was significantly enhanced by boosting with the same form of VLPs. Although the actual amount of Cap antigen in ELP-VLP was significantly less than that in VLP due to the significant difference in fusion tag length, immunization with the same dose of ELP-VLP induced even higher specific IgG response than with VLP. Importantly, the VN antibody titer induced by ELP-VLP was significantly (*p* < 0.01) higher than that by VLP. In addition, both Th1 (TNF-α, IFN-γ) and Th2 (IL-4) cytokine responses induced by ELP-VLP were also stronger than that by VLP, suggesting that ELP-VLP could promote the balanced Th1/Th2 response. Most importantly, immunization with ELP-VLP could provide the stronger protection against PCV2 challenge than with VLP. These data suggest that ELP-VLP can be developed further as a novel subunit vaccine against PCV2 infection.

## Conclusion

In this study, an ELPylation technology was established for easy preparation of PCV2 VLP vaccine. The established technology could be used for other VLP preparation and the ELP-VLP prepared could be developed further as a novel PCV2 subunit vaccine.

## Data Availability

All data and materials involved in this study are available if required.

## Abbreviations

PCV2: Porcine circovirus type 2
PCVAD: Porcine circovirus-associated disease
ELP: Elastin-like polypeptide
ITC: Inverse transition cycling
VLP: Virus-like particles
TEV: Tobacco etch virus
IPTG: Isopropyl β-D-thiogalactoside
TEM: Transmission electron microscopy
dpi: Days post infection
dpc: Days post challenge
ELISA: Enzyme-linked immunosorbent assay
VN: Virus neutralization
TCID_50_: 50% tissue infectious dose
FBS: Fetal bovine serum
IL-4: Interneukine-4
TNF-α: Tumor necrosis factor-α
IFN-γ: Interferon-γ
